# Industrial Applications of Selected JFS Articles

**DOI:** 10.1111/j.1750-3841.2010.01872.x

**Published:** 2010-10

Many papers this month emphasize the containment of cost as well as the enhancement of flavor and textures. This fits the current economic picture, as well as the need to make food go as far as possible. While the current feeling about the specter of starvation is mixed, (was Malthus right?) waste has a lot of other disadvantages, such as costing a lot of money for proper disposal.

## Vertical Integration in Fruit and Vegetable Processing Can Save Money and Reduce Processing Waste

A hypothesis paper explores the possibility of the use of byproducts from the trimming, slicing, and dicing of fresh fruits and vegetables in the race to make them more table-ready. Because the public likes fresh fruits and vegetables better than processed products, the convenience factor and the preference for “fresh” has caused a lot of producers to look hard at the different ways to produce a safer product. While the public prefers minimally processed foods, the growing concern about food safety must be dealt with. The authors of the paper “Antioxidant Enrichment and Antimicrobial Protection of Fresh-Cut Fruits Using Their Own Byproducts: Looking for Integral Exploitation” studied the possibilities and reports: The entire tissue of fruits and vegetables is rich in bioactive compounds, such as phenolic compounds, carotenoids, and vitamins. The fresh-cut fruit industry deals with the perishable character of its products and the large percentage of byproducts, such as peels, seeds, and unused flesh that are generated by different steps of the industrial process. In most cases, the wasted byproducts can present similar or even higher contents of antioxidant and antimicrobial compounds than the final produce can. In this context, this hypothesis article finds that the antioxidant enrichment and antimicrobial protection of fresh-cut fruits, provided by the fruit's own byproducts, could be possible.

The researchers have the following recommendations:

1. Optimize and scale-up the extraction procedures of bioactive constituents,
2. Evaluate the effect of the extraction procedure on the composition and activity of the obtained extracts, and identify the optimal application procedure and required doses to achieve both antimicrobial and antioxidant fortification without affecting sensorial acceptability.
3. If sensorial acceptability of the treated fruit is affected, the use of odor–flavor masking technologies could be contemplated, like incorporation of extracts in edible coatings, encapsulation technologies, and controlled release systems.

The research team pointed out that vertically integrating the process would reduce the amount of waste material produced, providing a lower waste agricultural system, and they believe profits for the products could rise. **R175-181**

## One Size May Not Fit All in Poultry Processes

The paper titled “Temperature and Bacterial Profile of Post Chill Poultry Carcasses Stored in Processing Combo Held at Room Temperature” presents a possibility that using a 2-tier processing method could both improve the safety of poultry and provide a lot of additional food to people who need it. The process might also reduce the expense to the processors, allowing them to hold selling price at a lower level. The work, which provides an alternative to a great deal of condemned poultry, may be especially attractive when economic times aren't booming.

The work followed good statistical practices, and found that the product could be designated for further processing before condemnation proceedings were instituted. Their conclusion was that “From the data collected in this study, the product is not microbiologically spoiled over the first 35 h. Therefore, data representing actual commercial processing conditions, it is proposed that whole carcasses designated as WIP be designated as safe for further processing for up to a maximum of 26 h after post chill at ambient room temperatures of 70 °F (21 °C) due to the relatively low increase in microbial growth and the overall combo being less than 55 °F.”**M515-520**

## Identifying Best Spices Uses Familiar Methods

In “Odor Active Compounds Content in Spices and Their Microencapsulated Powders Measured by SPME”, researchers studied the content of the major components of marjoram and thyme (linalool and thymol) in spices and microencapsulated powders containing the essential oils. Because changes occur in the content of essential oils both in the native spice and after processing, knowing how much of the oil is present can help in determining grades and provides the possibility of enhancing the products. The researchers note “Recently, solid-phase microextraction (SPME) in combination with gas chromatography (GC) has been applied increasingly often in the essential oil analyses of spices, including marjoram and thyme” and others. According to the researchers, “The application of SPME makes it possible to isolate a bigger amount of volatile compounds than in case of distillation.” Because essential oils and their volatile components show sensitivity to oxygen, light, temperature, and moisture, a rapid and efficient method can help to control their quality. The complexity of the compounds has made that difficult, but the combination of SPME and other methods appears to be effective in identifying the specific volatile compounds. The stability of volatile odorants, and essential oils, can be enhanced by microencapsulating the compounds. By identifying the content of special oils, a product can be developed to control release of encapsulated substances, improve taste, and extend shelf life of the final food product. **S441-445**

## Iron-Zinc Interactions Explored

In the paper “Dietary Ligands as Determinants of Iron–Zinc Interactions at the Absorptive Enterocyte”, researchers found some new wrinkles in the interaction of these 2 nutrients. According to the researchers, they have reported that iron and zinc interact at the enterocyte and influence the absorption of one another. They had reported, from previous research, that zinc noncompetitively inhibits iron uptake in Caco-2 cells, a widely accepted model of the absorptive enterocyte. However, the determinants of this interaction, such as the effect of dietary ligands, remain uncharacterized. They noted that “dietary ligands selectively chelate iron and zinc in definite stoichiometric proportions, and thus alter the bioavailability from food matrices. In this study, the team used common dietary ligands, such as ascorbic acid, phytic acid, tannic acid, tartaric acid, cysteine, histidine, and methionine to characterize iron, zinc uptake individually and in combination, using Caco-2 cells. Selective chelation of zinc-using cysteine decreased the magnitude of inhibition of iron uptake but could not reverse the inhibition. On the other hand, selective increase in iron uptake in the presence of methionine resulted in increased zinc uptake, rather than inhibition. Taken together, these *in vitro* results suggest that dietary ligands can modulate iron–zinc interaction and that zinc cannot competitively inhibit iron uptake.”

The difficulty of dealing with anemia—both iron and zinc—has called for information about the precise way that these 2 metals interact. The research team noted, “Understanding the nature and determinants of iron–zinc interactions during absorption assumes importance in this context of combined supplementation being recommended to simultaneously alleviate both deficiencies.” They further note: “Dietary ligands chelate iron and zinc in definite proportions and alter bioavailability of the mineral. Therefore, dietary ligands that selectively bind iron or zinc can be utilized to alter the availability of free metal ions in solution.” Further studies of this type may solve some of the problems of fortification of mixed systems. **H260-264**

## Apple a Day …

Whether or not apple components are believed to be good for you has varied over the years, but researchers with a positive attitude toward the fruit of Eve reported their findings on the subject in “Modified Apple Polysaccharides Could Induce Apoptosis in Colorectal Cancer Cells.” They start with the concept that dietary components can inhibit the progression of cancer, and proceeded to study the interactions between apple components and colorectal cancer cells. Three human colorectal cancer cell lines: SW-1116, HT-29, and Caco-2 were exposed to different concentrations of MAP (0% to 0.1%). Inhibition of cell proliferation was measured by MTT assay. DNA fragmentation was visualized by agarose-gel electrophoresis. The amount of apoptotic cells was assessed by flow cytometry, and protein level of caspase 3, 8, 9, Bax, and Bcl-2 was evaluated by Western blot. Modified apple polysaccharides appeared to show growth inhibition on cancer cells. There is a lot of information to be developed before one can claim anticancer activity for apple pie—but it's a good start. **H224-229**

## Antibrowning Agents Affect Color, Flavor

The “browning” reaction (Maillard) between products that include some protein, glucose, acid and enough water to trigger the reaction, produces unique flavors. Sometimes those flavors are too unique, and ways to better control the reaction are sought. The researchers that published the paper “Effect of Antibrowning Agents on Browning and Intermediate Formation in the Glucose–Glutamic Acid Model” were primarily concerned about the flavor and browning effect in soy products. They wanted to find which antibrowning agents could be used to control the production of glucose intermediates, such as 3-deoxyglucosone (3-DG) and hydroxymethylfurfural (HMF). The initial antibrowning capacity was measured in the following order: pentasodium tripolyphosphate< citric acid and oxalic acid < cysteine and glutathione < sodium sulfite. The data showed that antibrowning agents, such as pentasodium tripolyphosphate, citric acid, and oxalic acid, maintained antibrowning capacities during storage at both 4 and 30 °C. However, both cysteine and glutathione was reduced with storage time, especially in the air. These compounds were produced most abundantly in the presence of sodium sulfite, and the yields were not related significantly to the degree of browning. Citric acid and oxalic acid were identified as the most effective in inhibitors of browning and intermediates, even during storage in air at 30 °C. Soybean products used to season Chinese, Korean, and Japanese cuisine have a complicated taste profile, which can be significantly changed by the production of these intermediaries, to the level of spoiling the taste profile and making the products unsalable. **C678-683**

